# Supplementary material for: A Likelihood Approach for Real-Time Calibration of Stochastic Compartmental Epidemic Models
Source: PLoS Comput Biol. 2017 Jan 17;13(1):e1005257. doi: 10.1371/journal.pcbi.1005257 (PMC5240920; doi:10.1371/journal.pcbi.1005257)
Supplement: S1 File — (TAR.GZ) [file pcbi.1005257.s014.tar.gz › HSPH_Online-SI-Revision/PF08/n1-severe/n1-severe_PF_table8.pdf]

1	R0	{1.51081, 13.7078}	{1.64266, 14.6626}	{{1.23706, 8.24622}, {2.29768, 20.}}	{20.7578, 28.7924}	inf	54.	28.0201	{2.37801, 59.1082}	58.7233	0.575838	newly	28.	11.04	{1., 23.}	62.8571	0.665367	Reff	0.70857	0.649414	{0.34266, 0.878009}	23.6286	ar	656.	749.48	{534., 983.}	24.253
2	R0	{1.73182, 11.3873}	{1.72793, 14.5647}	{{1.2732, 8.17083}, {2.25131, 20.}}	{17.8707, 40.1292}	inf	39.	35.0594	{4.9543, 62.7952}	56.3916	0.379635	newly	19.	13.83	{3., 26.}	46.4737	0.359856	Reff	0.680605	0.589886	{0.358164, 0.791177}	22.4733	ar	653.	798.86	{561., 991.}	28.3828
3	R0	{1.50449, 10.3474}	{1.6512, 14.6144}	{{1.23264, 8.13391}, {2.20018, 20.}}	{21.5123, 50.2877}	inf	38.	32.5545	{4.75714, 62.7126}	56.5497	0.381062	newly	22.	13.27	{2., 26.}	51.5909	0.428748	Reff	0.664985	0.646519	{0.3476, 0.884291}	23.524	ar	651.	755.3	{542., 994.}	24.7619
4	R0	{1.63493, 9.1955}	{1.98049, 15.1394}	{{1.38934, 7.57343}, {2.74304, 20.}}	{28.1864, 71.4872}	inf	17.	46.4337	{10.226, 92.6426}	188.61	0.425771	newly	9.	18.35	{8., 32.}	113.667	0.300915	Reff	0.614734	0.636635	{0.392121, 0.932646}	27.139	ar	663.	862.36	{606., 1040.}	33.1704
5	R0	{1.53822, 8.05553}	{1.8688, 15.2954}	{{1.26717, 9.74891}, {2.52688, 20.}}	{28.9006, 92.8228}	inf	26.	40.6281	{8.52867, 87.5359}	87.5512	0.281927	newly	17.	17.05	{5., 36.}	53.3529	0.252911	Reff	0.642976	0.754876	{0.4773, 1.02884}	30.9533	ar	621.	803.47	{558., 1009.}	35.0709
6	R0	{1.69374, 17.0793}	{1.63462, 14.5027}	{{1.19173, 7.24169}, {2.13306, 20.}}	{19.6539, 23.9367}	inf	76.	31.2899	{1.31386, 66.1557}	60.6552	0.695822	newly	35.	12.5	{1., 26.}	64.4571	0.729516	Reff	0.753714	0.633973	{0.372383, 0.850938}	21.9499	ar	662.	734.52	{490., 967.}	24.2477
7	R0	{1.42179, 11.8031}	{1.63866, 14.5587}	{{1.2211, 8.99124}, {2.25851, 19.7549}}	{23.0666, 35.3608}	inf	20.	23.0153	{3.66508, 48.2527}	67.3074	0.375389	newly	9.	9.22	{1., 19.}	59.3333	0.39275	Reff	0.720848	0.613638	{0.333337, 0.834292}	23.271	ar	517.	748.92	{539., 1000.}	46.6306
8	R0	{1.46683, 6.95875}	{1.93238, 14.6729}	{{1.24539, 8.28074}, {2.71376, 20.}}	{38.029, 114.881}	inf	0.1	43.1192	{10.2715, 84.3889}	43 019.2	2.50105	newly	0.1	17.67	{5., 34.}	17 570.	2.13899	Reff	0.50899	0.631253	{0.375809, 0.916103}	38.0395	ar	653.	847.29	{537., 1043.}	35.827
9	R0	{1.34525, 11.505}	{1.67775, 14.6644}	{{1.22244, 8.12155}, {2.24928, 19.9569}}	{28.8641, 39.9146}	inf	36.	27.8694	{3.74024, 60.9956}	63.3893	0.48595	newly	22.	10.71	{2., 23.}	58.4091	0.542556	Reff	0.768138	0.606984	{0.355503, 0.848604}	25.7392	ar	532.	767.	{496., 987.}	47.2594
10	R0	{1.77958, 14.372}	{1.71555, 15.2828}	{{1.26114, 9.91502}, {2.30372, 20.}}	{18.6256, 23.6756}	inf	31.	27.754	{4.66567, 54.8212}	56.5015	0.34904	newly	16.	10.29	{2., 20.}	50.3125	0.385757	Reff	0.619294	0.570674	{0.297367, 0.790224}	23.2361	ar	671.	795.05	{561., 1006.}	25.9538
11	R0	{1.60334, 19.9548}	{1.593, 13.829}	{{1.19719, 6.67068}, {2.2103, 20.}}	{19.0326, 30.7529}	inf	70.	18.2356	{0.0781908, 43.9551}	74.4662	1.05102	newly	28.	7.44	{0., 18.}	73.5714	1.03847	Reff	0.80167	0.631251	{0.385391, 0.828766}	22.912	ar	617.	719.87	{486., 950.}	26.4749
12	R0	{1.68623, 7.58585}	{1.88803, 13.7799}	{{1.27627, 8.51468}, {2.64373, 18.7523}}	{25.5551, 86.6686}	inf	7.	25.0646	{2.44235, 50.2721}	281.179	0.519823	newly	7.	10.55	{2., 23.}	95.2857	0.342477	Reff	0.612101	0.544062	{0.274034, 0.812348}	29.6326	ar	640.	702.27	{414., 910.}	25.7922
13	R0	{1.82689, 18.0022}	{1.67343, 15.5801}	{{1.19387, 7.58978}, {2.31252, 20.}}	{20.6798, 20.0971}	inf	56.	33.4807	{4.25362, 74.0059}	53.8091	0.461569	newly	15.	12.86	{3., 26.}	48.5333	0.340639	Reff	0.668642	0.618946	{0.313444, 0.875003}	25.8128	ar	673.	760.39	{489., 995.}	25.7578
14	R0	{1.75911, 9.16883}	{1.83768, 14.0763}	{{1.23008, 8.91444}, {2.39017, 20.}}	{22.354, 60.5911}	inf	25.	50.7321	{17.511, 104.641}	116.696	0.298488	newly	15.	22.43	{9., 36.}	69.8	0.227097	Reff	0.656148	0.756183	{0.485635, 1.08848}	29.1825	ar	661.	806.26	{543., 1007.}	30.2632
15	R0	{1.46168, 11.3859}	{1.75795, 14.2973}	{{1.23551, 8.2453}, {2.3832, 19.9475}}	{28.4801, 38.0442}	inf	9.	18.4728	{1.34, 42.1172}	147.569	0.463902	newly	3.	7.49	{0., 16.}	192.333	0.5564	Reff	0.654833	0.570771	{0.333601, 0.805644}	26.656	ar	556.	788.29	{552., 1005.}	44.0306
16	R0	{1.53577, 11.7581}	{1.81887, 13.7887}	{{1.21457, 8.33653}, {2.52006, 19.0269}}	{27.724, 33.1424}	inf	31.	29.0145	{2.61494, 70.4854}	69.9167	0.4646	newly	13.	11.94	{1., 27.}	66.1538	0.435549	Reff	0.664988	0.585708	{0.285507, 0.861814}	29.18	ar	625.	679.29	{364., 896.}	26.4784
17	R0	{1.70698, 9.72924}	{1.84964, 13.9157}	{{1.21937, 7.48339}, {2.46976, 18.4749}}	{21.7639, 52.4922}	inf	8.	28.1508	{2.24985, 58.6513}	279.882	0.585844	newly	4.	11.21	{1., 23.}	209.25	0.520027	Reff	0.571838	0.552512	{0.302281, 0.821198}	26.8261	ar	675.	692.92	{361., 889.}	21.84
18	R0	{1.5973, 14.931}	{1.78442, 15.0486}	{{1.23118, 9.1641}, {2.37941, 20.}}	{25.1905, 22.3821}	inf	59.	35.3816	{13.8318, 62.2901}	49.7724	0.336019	newly	28.	15.06	{7., 26.}	51.4286	0.351455	Reff	0.967964	0.844364	{0.527859, 1.18258}	24.1679	ar	632.	760.99	{481., 976.}	30.9193
19	R0	{1.87468, 17.004}	{1.7046, 15.5292}	{{1.23534, 8.37368}, {2.29993, 20.}}	{20.7609, 19.7443}	inf	48.	21.428	{3.27949, 40.0225}	62.1468	0.551491	newly	12.	8.17	{1., 18.}	53.25	0.447078	Reff	0.684258	0.567053	{0.29971, 0.799206}	25.3823	ar	692.	779.25	{540., 992.}	23.6315
20	R0	{1.84715, 12.531}	{1.71071, 15.1888}	{{1.25813, 7.71423}, {2.33228, 20.}}	{20.3246, 37.5722}	inf	9.	21.3578	{1.37545, 46.0505}	173.48	0.51479	newly	3.	8.02	{0., 19.}	201.333	0.546827	Reff	0.676057	0.567195	{0.29725, 0.789352}	24.4498	ar	661.	779.61	{520., 972.}	25.2239
21	R0	{1.75938, 13.5959}	{1.70325, 13.5165}	{{1.14292, 8.09734}, {2.31174, 18.4377}}	{21.188, 26.6336}	inf	38.	25.6527	{2.29065, 56.5873}	61.3867	0.508907	newly	12.	10.6	{1., 22.}	61.5	0.41025	Reff	0.777646	0.617193	{0.325759, 0.861814}	28.9881	ar	620.	635.42	{326., 858.}	26.0194
22	R0	{1.63456, 10.7469}	{1.71427, 15.2768}	{{1.24453, 9.53738}, {2.31524, 20.}}	{20.4852, 51.8417}	inf	11.	25.9966	{2.46058, 50.0379}	168.373	0.495222	newly	7.	9.83	{1., 21.}	89.	0.435271	Reff	0.568827	0.581179	{0.311519, 0.831714}	28.1159	ar	654.	776.35	{487., 979.}	28.7385
23	R0	{1.62702, 19.1392}	{1.76017, 14.546}	{{1.27088, 7.83487}, {2.29686, 20.}}	{21.8578, 25.2686}	inf	42.	30.01	{6.00251, 70.2453}	57.5673	0.404029	newly	16.	13.02	{4., 28.}	57.	0.356587	Reff	0.715889	0.746395	{0.502264, 1.01723}	24.045	ar	600.	765.37	{537., 972.}	34.0783
24	R0	{1.70466, 12.1202}	{1.80397, 14.4926}	{{1.26239, 8.31755}, {2.52245, 19.8079}}	{22.8994, 33.0911}	inf	15.	21.7189	{1.53959, 53.9419}	104.992	0.430497	newly	6.	9.26	{1., 23.}	108.	0.428456	Reff	0.685273	0.687135	{0.453599, 0.888049}	22.2868	ar	613.	779.77	{557., 982.}	32.8597
25	R0	{1.61735, 11.3169}	{1.6267, 14.6951}	{{1.22375, 7.77833}, {2.0902, 20.}}	{17.3157, 41.0723}	inf	17.	16.3226	{1.25372, 35.4752}	68.9774	0.45578	newly	11.	6.61	{0., 13.}	60.2727	0.556629	Reff	0.533726	0.584273	{0.367967, 0.768381}	25.85	ar	681.	752.15	{538., 947.}	20.8326
26	R0	{1.56977, 12.3035}	{1.74019, 13.1902}	{{1.15921, 4.98002}, {2.31231, 19.2972}}	{25.3415, 35.0748}	inf	23.	20.9361	{0.428047, 57.487}	76.0494	0.590975	newly	15.	8.4	{0., 21.}	65.8667	0.689424	Reff	0.758199	0.613158	{0.321247, 0.849895}	30.8766	ar	594.	638.94	{360., 864.}	30.7811
27	R0	{1.46317, 5.8298}	{1.86035, 14.4964}	{{1.25558, 7.53886}, {2.42731, 20.}}	{32.3704, 150.327}	inf	0.1	35.5093	{5.44902, 88.6875}	35 409.3	2.38102	newly	0.1	14.49	{3., 32.}	14 394.	1.98998	Reff	0.605752	0.650957	{0.418875, 0.917191}	26.2882	ar	586.	824.88	{554., 1015.}	43.1741
28	R0	{1.70002, 14.9806}	{1.9154, 15.0728}	{{1.27501, 6.61339}, {2.74944, 20.}}	{25.9616, 23.6891}	inf	50.	48.2151	{13.7455, 107.859}	59.5922	0.296923	newly	31.	20.7	{7., 42.}	50.4516	0.323214	Reff	0.683408	0.850433	{0.498358, 1.22793}	36.3769	ar	652.	813.3	{566., 1019.}	31.2454
29	R0	{1.52729, 16.1014}	{1.62325, 14.431}	{{1.20248, 7.24024}, {2.27543, 20.}}	{20.7465, 24.2758}	inf	25.	18.831	{1.57741, 39.7405}	61.6926	0.52252	newly	10.	7.37	{0., 16.}	55.3	0.497836	Reff	0.720881	0.613575	{0.340497, 0.8224}	22.7477	ar	592.	732.18	{513., 980.}	30.5439
30	R0	{1.72795, 13.5635}	{1.74616, 14.5435}	{{1.27182, 8.14954}, {2.29244, 19.7926}}	{19.1904, 29.0177}	inf	17.	23.3494	{1.28357, 50.0264}	94.0209	0.460711	newly	2.	8.82	{1., 19.}	366.	0.636726	Reff	0.60651	0.574679	{0.324188, 0.793845}	24.3932	ar	668.	794.41	{552., 998.}	26.6542
31	R0	{1.51591, 18.1544}	{1.66714, 13.0315}	{{1.21569, 6.08006}, {2.35463, 20.}}	{22.4297, 31.5061}	inf	38.	25.4228	{0.262981, 54.8083}	67.6544	0.722659	newly	24.	10.53	{0., 25.}	62.875	0.804222	Reff	0.858005	0.639651	{0.350231, 0.843284}	26.7976	ar	541.	746.48	{507., 984.}	41.305
32	R0	{1.4282, 10.3534}	{1.71874, 14.4976}	{{1.21098, 7.24532}, {2.48064, 19.9888}}	{27.4128, 51.7341}	inf	37.	31.3947	{4.84306, 66.5171}	58.1508	0.423797	newly	27.	12.54	{2., 26.}	56.6667	0.520936	Reff	0.854064	0.612179	{0.307243, 0.845232}	29.4711	ar	521.	777.86	{534., 1001.}	50.8407
33	R0	{1.56693, 11.3972}	{1.972, 14.3907}	{{1.32854, 8.43465}, {2.56783, 18.5596}}	{32.8583, 35.8274}	inf	21.	36.8155	{11.8088, 84.9485}	95.0922	0.254483	newly	13.	16.25	{7., 36.}	59.	0.213206	Reff	0.518654	0.773322	{0.513085, 1.14277}	51.914	ar	695.	816.46	{526., 985.}	28.1899
34	R0	{1.50675, 19.8823}	{1.6521, 14.1757}	{{1.18279, 7.90686}, {2.24126, 19.9186}}	{20.4441, 28.8267}	inf	31.	16.8335	{1.19063, 38.3708}	63.562	0.599059	newly	10.	7.11	{0., 16.}	65.1	0.58288	Reff	0.729267	0.625847	{0.382384, 0.821733}	20.9667	ar	587.	759.36	{520., 987.}	34.3509
35	R0	{1.72889, 7.76068}	{2.10468, 14.7894}	{{1.34659, 8.30808}, {2.97585, 20.}}	{31.2951, 96.4502}	inf	3.	37.8858	{9.05859, 66.1334}	1162.86	1.01005	newly	3.	15.98	{7., 26.}	433.333	0.662269	Reff	0.605112	0.678823	{0.422253, 0.958394}	28.5796	ar	650.	853.38	{585., 1020.}	36.2831
36	R0	{1.62644, 4.12303}	{1.98727, 13.0174}	{{1.27231, 7.03829}, {2.78007, 18.9406}}	{33.1872, 216.109}	inf	0.1	18.9747	{0.939891, 44.0484}	18 876.8	1.98398	newly	0.1	7.71	{1., 17.}	7626.	1.65118	Reff	0.566001	0.500356	{0.235238, 0.826921}	36.8191	ar	652.	716.14	{425., 913.}	29.5644
37	R0	{1.70878, 15.8072}	{1.66541, 14.6723}	{{1.23785, 7.92723}, {2.3069, 20.}}	{21.8504, 25.2942}	inf	40.	15.1704	{1.30616, 35.2454}	66.3432	0.736231	newly	11.	5.73	{0., 14.}	63.1818	0.637151	Reff	0.659589	0.580276	{0.305853, 0.794306}	24.1815	ar	674.	753.7	{521., 997.}	24.1573
38	R0	{1.58967, 6.80568}	{1.99783, 14.1042}	{{1.32167, 7.44114}, {2.7648, 20.}}	{32.8586, 110.723}	inf	5.	36.7948	{8.54421, 85.0947}	637.574	0.725451	newly	3.	15.77	{5., 32.}	428.333	0.605824	Reff	0.62474	0.651326	{0.419859, 0.9347}	25.901	ar	610.	831.85	{538., 1022.}	41.9033
39	R0	{1.344, 8.19498}	{1.64133, 14.8306}	{{1.20283, 7.72083}, {2.34857, 20.}}	{26.759, 85.4031}	inf	14.	27.377	{4.10349, 52.1265}	130.416	0.408555	newly	14.	10.91	{2., 21.}	50.7857	0.391738	Reff	0.684096	0.621332	{0.301966, 0.83079}	22.1878	ar	555.	742.25	{496., 1000.}	38.9201
40	R0	{1.61509, 7.90825}	{1.78165, 13.3072}	{{1.11228, 6.74711}, {2.46533, 18.4249}}	{26.3615, 73.6802}	inf	13.	28.3348	{1.9646, 57.4407}	161.83	0.49234	newly	9.	11.73	{1., 27.}	89.	0.414112	Reff	0.7171	0.590567	{0.301938, 0.865003}	29.4541	ar	581.	657.8	{317., 889.}	34.1962
41	R0	{1.60017, 5.90605}	{1.99475, 14.5041}	{{1.26811, 8.72398}, {2.776, 19.3461}}	{33.1568, 147.75}	inf	6.	35.4164	{7.49948, 67.9691}	497.203	0.673084	newly	6.	14.76	{4., 29.}	161.333	0.382998	Reff	0.547258	0.59035	{0.341867, 0.876074}	32.2017	ar	661.	857.91	{580., 1039.}	35.0121
42	R0	{1.64436, 15.1337}	{1.71412, 13.4139}	{{1.13239, 6.05643}, {2.27582, 19.1171}}	{23.4707, 26.9513}	inf	40.	22.9586	{0.944861, 50.0365}	64.3621	0.648564	newly	15.	9.21	{0., 19.}	63.8	0.613658	Reff	0.779427	0.637803	{0.357996, 0.906829}	30.6216	ar	640.	622.82	{324., 872.}	28.0094
43	R0	{1.48917, 11.917}	{1.5402, 13.9181}	{{1.17294, 6.11197}, {2.02373, 20.}}	{18.1047, 38.8193}	inf	13.	17.2415	{0.166864, 45.3957}	104.597	0.637823	newly	9.	6.56	{0., 16.}	67.5556	0.692591	Reff	0.775858	0.638295	{0.417953, 0.807322}	19.6519	ar	551.	697.64	{473., 902.}	31.9528
44	R0	{1.65577, 7.89317}	{1.91651, 15.0144}	{{1.26473, 7.42378}, {2.74609, 20.}}	{28.1946, 94.9673}	inf	7.	47.6081	{12.5562, 105.723}	580.115	0.722991	newly	5.	20.22	{7., 39.}	306.4	0.535534	Reff	0.738473	0.750132	{0.460755, 1.08722}	27.4574	ar	576.	825.13	{583., 1029.}	46.1337
45	R0	{1.6909, 12.3275}	{1.80008, 15.0007}	{{1.19871, 7.85963}, {2.52566, 20.}}	{24.3166, 37.6712}	inf	23.	37.128	{7.17536, 81.9409}	96.5819	0.328084	newly	8.	15.37	{4., 32.}	115.375	0.358707	Reff	0.654378	0.731683	{0.440171, 1.0047}	31.5231	ar	675.	792.21	{528., 1004.}	28.7481
46	R0	{1.62351, 16.0698}	{1.60534, 14.436}	{{1.1873, 7.66141}, {2.20953, 20.}}	{20.6433, 23.904}	inf	51.	25.3758	{2.75363, 51.099}	53.9208	0.562873	newly	13.	10.63	{1., 21.}	49.9231	0.415053	Reff	0.899425	0.643923	{0.36578, 0.855454}	28.9033	ar	591.	725.06	{465., 965.}	30.7513
47	R0	{1.52372, 4.38599}	{2.10499, 14.851}	{{1.46013, 10.4985}, {2.71993, 19.5473}}	{41.3155, 238.879}	inf	0.1	47.4873	{13.6268, 81.7887}	47 387.3	2.56756	newly	0.1	19.6	{6., 34.}	19 500.	2.19704	Reff	0.582061	0.568371	{0.361557, 0.822898}	26.8664	ar	618.	899.9	{693., 1040.}	48.0971
48	R0	{1.44306, 11.788}	{1.76532, 13.7404}	{{1.20153, 5.62573}, {2.35669, 19.6459}}	{29.6048, 39.7622}	inf	22.	30.1435	{3.31373, 62.7027}	83.1431	0.359318	newly	12.	13.81	{2., 28.}	69.25	0.374191	Reff	0.722973	0.713242	{0.428405, 0.971591}	24.9389	ar	534.	775.65	{502., 1004.}	48.1816
49	R0	{1.63624, 11.3893}	{1.82756, 13.5785}	{{1.16434, 8.39294}, {2.39393, 18.6082}}	{25.3189, 32.7915}	inf	22.	21.8451	{2.64784, 52.4118}	67.3044	0.415899	newly	9.	9.16	{1., 22.}	66.6667	0.416694	Reff	0.623407	0.548786	{0.298889, 0.791848}	28.4196	ar	423.	684.23	{389., 876.}	26.5117
50	R0	{1.46113, 11.1322}	{1.65361, 13.9726}	{{1.22178, 7.6105}, {2.36219, 20.}}	{22.5641, 42.3148}	inf	28.	30.5263	{2.45889, 63.8506}	68.9366	0.413034	newly	17.	12.38	{2., 23.}	47.0588	0.390195	Reff	0.831383	0.634951	{0.324685, 0.868116}	25.9981	ar	501.	751.49	{525., 1004.}	[illegible]8403
